# Investigating changes within the handling system of the largest semi-captive population of Asian elephants

**DOI:** 10.1371/journal.pone.0209701

**Published:** 2019-01-31

**Authors:** Jennie A. H. Crawley, Mirkka Lahdenperä, Martin W. Seltmann, Win Htut, Htoo Htoo Aung, Kyaw Nyein, Virpi Lummaa

**Affiliations:** 1 Department of Biology, University of Turku, Turku, Finland; 2 Myanma Timber Enterprise, Yangon, Myanmar; Bowling Green State University, UNITED STATES

## Abstract

The current extinction crisis leaves us increasingly reliant on captive populations to maintain vulnerable species. Approximately one third of Asian elephants (*Elephas maximus*) are living in semi-captive conditions in range countries. Their relationship with humans stretches back millennia, yet elephants have never been fully domesticated. We rely on the expertise of traditional handlers (mahouts) to manage these essentially wild animals, yet this profession may be threatened in the modern day. Here, we study the handling system of semi-captive timber elephants in Myanmar; the largest global semi-captive population (~5 000). We investigate how recent changes in Myanmar may have affected the keeping system and mahout-elephant interactions. Structured interviews investigated changes to mahout attitude and experience over the last two decades, as perceived by those who had worked in the industry for at least 10 years (n = 23) and as evaluated in current mahouts (n = 210), finding mahouts today are younger (median age 22yrs), less experienced (median experience 3yrs), and change elephants frequently, threatening traditional knowledge transfer. Mahout-elephant interactions manifested as 5 components (‘job appreciation’; ‘experience is necessary’; ‘human-elephant interaction’; ‘own knowledge’; ‘elephant relationship’), according to Principal Components Analysis. Experienced mahouts and mahouts of bulls and younger elephants were more likely to agree that ‘experience is necessary’ to be a mahout. Mahouts with difficult elephants scored lower on ‘human-elephant interaction’ and a mahout’s perception of their ‘own knowledge’ increased with more experience. Our finding of change in terms of mahout experience, age and commitment in the largest semi-captive elephant population suggests need for formal training and assessment of impacts on elephant welfare; these are findings applicable to thousands of elephants under similar management.

## Introduction

The current extinction crisis is leaving more species reliant on human management to conserve vulnerable populations [1]. One important intervention is the maintenance of ex-situ captive populations for conservation purposes, such as species specific education, research and breeding programmes and conservation of the gene pool for eventual translocations or reintroductions [2]. A major challenge for ex-situ conservation is to minimise human-directed selection whilst in captivity, to prevent captive populations undergoing changes in temperament [3], behaviour [4], or reproduction [5]. This can be driven by selection in their captive environment, which is often at odds with their natural habitat [6]. Such problems are lessened when populations are kept in more natural environments, such as semi-captive conditions. Semi-captive animals range freely in their natural environment, with varying levels of veterinary care, diet supplementation and shelter. Semi-captive populations are common, existing in primates [7,8], birds [9,10], ungulates [11,12], and elephants [13,14].

Like many far ranging and browsing species, elephants do not cope well in zoos [15,16], often showing altered behaviour, reduced reproduction, and survival compared to wild and semi-captive elephants living in their range countries [17,18]. If managed appropriately, semi-captive populations could therefore be a vital reservoir for the endangered Asian elephant (*Elephas maximus*), especially as they constitute approximately one third (~15 000) of the remaining global population of Asian elephants, compared to <1 000 in zoos [19]. Despite their large value for conservation, many current semi-captive elephant populations are not primarily maintained for conservation purposes, but are instead used for work in logging, transport, tourist camps or temples. Semi-captive elephants have been maintained as draught animals for millennia throughout Asia, but as their reproduction has always been largely independent of humans, they have never been selectively bred to domestication [19]. Due to lack of domestication, elephant management in these semi-captive populations has instead relied on the expertise of specialised handlers (mahouts), which has accumulated over many generations [20].

Elephant management worldwide has undergone major transformations in recent decades. First, many elephant managers are moving towards more “hands-off” techniques seen in the introduction of protected contact in zoos and less intensive elephant tourism in Thailand, strategies aiming to minimise human induced stress [21,22]. Second, the traditional mahouts who care for semi-captive elephant populations in range countries have faced changes to the economic importance and cultural appreciation of elephants and their profession, as well as pressure from elephant welfare groups. Mahouts traditionally learn handling skills from a young age, working with the same elephant for many years, sometimes decades [23]. Experienced mahouts build up invaluable knowledge [24], such as the hundreds of plants in their elephant’s diet [25], or the ability to read subtle behavioural signals for example through their vocalisations [26]. Although there has been some literary documentation of elephant care such as the *gajaśāstra* (Sanskrit writings on elephant science), the vast majority of knowledge is transferred through observation and apprenticeship. As this cycle is reliant on the availability of experienced mahouts, it is vulnerable to disruption [20]. We must understand how this process fares in the modern day, as it is linked to the health, safety and welfare of both the elephants and the mahouts caring for these large and essentially wild animals. Traditional handling also relies on a taming procedure which has been criticised in relation to elephant welfare, and must also be addressed in the dialogue of modern handling [21].

Social change has been found to disrupt the mahout profession over recent decades in parts of Asia, with reduced employment opportunities, salaries unable to compete with growing industries [27], and lessened cultural appreciation observed in India, Nepal, Laos and Thailand [28–30]. There have been many valuable studies of elephant care in these countries, but these were often small scale (<30 elephants; [20,26,28,31]) or focused on specific aspects of the job (e.g. associated risks [32,33]). Semi-captive Asian elephants are scattered across sectors (tourism, logging, sanctuaries, temples), and often lack centralised management, making large-scale study difficult [29]. The large scale studies that have been carried out generally found that there were few mahouts from a traditional handling background and few employment opportunities [30,34,35]. In India, these changes have been linked to mahouts spending less time with their elephants, and changing elephants more frequently, jeopardising elephant care [34]. In Laos, there is an ageing population of forestry mahouts, whereas mahouts in the tourism industry are often young and inexperienced [30]. Myanmar is home to the largest semi-captive Asian elephant population (~5 000), being the only country still extensively employing elephants in the timber industry and considered by many to be one of the last strong-holds of traditional mahout knowledge [19]. The mahout system in Myanmar therefore has implications on the health, demography and wellbeing of thousands of elephants, yet little is known of how it fares in the modern day. Recent political shifts in Myanmar have been coupled with increased urbanisation and social freedom, which have improved communication and access to remote areas [36]. These changes have likely affected the mahout profession in Myanmar as in other countries across Asia [24], and an exploration of any changes and subsequent effects is long overdue.

Here we investigate the largest mahout system in the world, using a questionnaire approach to collect information otherwise unavailable, to address our hypothesis that the mahout system in Myanmar has undergone shifts in recent years. We first examine recent changes to mahout demography, attitude and commitment through questionnaires posed to 23 men with at least 10 years expertise working with timber elephants in Myanmar (as a vet, mahout, or head mahout). We then report detailed questionnaires answered by 210 handlers working within elephant camps today to assess current mahout demography, experience and attitudes surrounding their job. Findings evaluate current mahouts in relation to the assumed traditional system to assess potential implications for elephant welfare. Our conclusions are applicable to one third of this species under human management, and more widely informative as human-managed populations come to play an increasing role in conservation.

## Materials and methods

### Study population

Here we study the keeping system of elephants owned by Myanma Timber Enterprise (MTE), who own and manage half of the country’s semi-captive elephants (~2 700). The elephants are classed as semi-captive: when not working they are released into the forest to forage and interact with other semi-captive and wild elephants, reproducing without human interference. All elephants have an ID number and corresponding logbook detailing demographic and health information. The MTE elephants are rested during the hot season (March-May), their work hours and tonnage are restricted according to season, the manner of labour, and their condition. All MTE elephants are retired by age 55 and pregnant females are rested from half-way through pregnancy until their calf is one year old, whereupon they return to light tasks. Calves are kept with their mother, with little human contact, until weaning at 4–5 years, at which point they are tamed.

Each elephant has one mahout responsible for their care in a working group of around 6 elephants. Each working group is overseen by a head mahout (sin-gaung) who does not have their own elephant, but is instead responsible for the whole group. A sin-oke coordinates a region of around 100 elephants, and an MTE veterinarian examines each elephant at least once a month. Taming takes place over one month in the cold season, aiming to habituate calves to humans, and for the calf to accept a rider. The calf is surrounded by mahouts whilst restrained using a cradle (breast-band), and the mahouts spend a lot of time rubbing the calf whilst singing to them to allow them to become familiar with human contact (see [13,37] for more details).Parts of this procedure are considered to cause psychological and physical trauma with many experts favouring methods based on positive reinforcement with gradual introduction to humans [38].

### Participants

This study involved human participants, and ethical approval was granted from the University of Turku's ethical board. Informed consent was obtained from each participant during spoken interviews. To assess changes in the mahout profession we created two questionnaires aimed at different subject groups. The questionnaire approach can have limitations but it was deemed the most appropriate method in this case, to collect historical and current mahout information otherwise non-existent and difficult to obtain, following guidelines suggested by Young *et al*. [39] and similar to Bansiddhi *et al*. [22]. All participants were recruited from MTE elephant camps during routine data collection from the elephants and participants were generally familiar with the questionnaire interview process. The questionnaires were created to address our hypotheses, the first “expert questionnaire” directed at those with at least 10 years of experience working with MTE mahouts and their elephants, to record any changes perceived since they began working. All 23 respondents were men (usual for these professions): 11 sin-gaung, five veterinarians, five mahouts, and two sin-oke, collectively referred to as ‘experts’ from here on. The second “mahout questionnaire” was directed at 210 current handlers, again all men: 188 mahouts, 17 sin-gaung, three sin-oke, and two apprentices. These latter respondents may also have been included in the “expert” sample if experienced enough. We present results of apprentices together with mahouts, and sin-oke with sin-gaung as head mahouts for reporting ease, with mahouts and head mahouts reported separately where questions apply to both (some questions, e.g. own elephant information, concern only mahouts). Questions for the mahout questionnaire were trialled in 2016, followed by a restructuring process to improve the overall clarity and informative power of the questionnaire, leading to a more extensive questionnaire in 2017 and 2018.

### Questionnaires

Questionnaires were translated from English into Burmese by a bilingual native speaker with extensive knowledge on the handling system and filled in during spoken interviews conducted by 7 interviewers in Burmese (to aid illiterate interviewees and obtain informed consent). Mahout interviews were conducted over 5 days in spring 2016 (*n* = 36), 8 days in spring 2017 (*n* = 108) (alongside expert interviews (*n* = 23)), and 8 days in spring 2018 (*n* = 66) in the Sagaing region of Myanmar. Questions (shown in **S1 Table**) were either open ended, multiple choice, or on a 1–5 Likert scale, and were read to the interviewee as shown, but explained further if required. Ages and time durations should be treated as estimates as formal records are rare. Reported averages refer to median values unless stated otherwise, and percentages are rounded to the nearest integer. All statistical analyses were conducted in R (version 3.4.0, [40]).

#### Expert questionnaires

The expert questionnaire consisted of 12 questions covering perceived changes in mahout demographics and attitudes since the interviewee began working (see **S1 Table** for full questionnaire) with multiple choice options of directional change (e.g. *younger/ older*), *unsure* or *no change*. We report answers as percentages and tested the significance of directional changes (excluding *unsure*/*no change*) using Pearson’s Chi-squared test. Final questions asked experts to elaborate on changes, and estimate when changes occurred.

#### Mahout questionnaires

Mahout questionnaires investigated whether current mahouts differ from the assumed traditional system. Basic details were asked of all 210 handlers (10 questions), whilst 178 answered an additional 19 questions, in the collection years 2017 & 2018 (see **S1 Table**). Questions covered personal details (e.g. age, marital status, family history), level of expertise (e.g. number of elephants, time with current elephant/as mahout/apprentice, taming experience), and opinions (e.g. elephant sex preference, job commitment). Questions on taming determined mahout attitudes towards traditional and novel techniques. We tested mahout openness to reward-based taming in relation to their age and experience, by fitting a *polr* model from the MASS package [41], with the response variable of mahout openness (ordinal, 1 = Not possible, 2 = Sometimes possible, 3 = Possible), and two explanatory variables of mahout age (continuous: 15–59) and total number of years working with elephants (continuous: 0.25–43 years). To explore knowledge on elephant behaviour, we chose two behaviours thought to indicate difficult behaviour: “shaking head on approach” and “using trunk to hit ground/swing at others”, as described in the literature to be threatening or agonistic [42,43]. We asked mahouts if their elephant displayed these behavioural signals and how they interpret this lack or display of behaviour, to see if they interpreted these as we would expect (that these signals indicate difficult behaviour).

Final questions of the mahout questionnaire asked agreement with 22 statements surrounding elephants and their job on a 1–5 Likert scale (*definitely; quite; unsure; not really; definitely not*). Using Principal Components Analysis (PCA), we reduced these statements to fewer dimensions to address further questions (similar to Ward and Melfi, 2015, [44]. We used the *Principal* function (*psych* package: Revelle, 2017, [45]) with *varimax* rotation, as we expected statements to correlate. We removed four statements as >97% answered *definitely* (‘elephants are interesting’; ‘beautiful’, ‘feel pain’ and ‘interviewee wants to learn more about elephants’), as well as ‘elephants are dangerous’ which correlated <0.3 with other statements (as in Ward and Melfi, 2015, [44]). All remaining statements correlated >0.3 with at least one other, and no more than 0.9, indicating absence of multi-collinearity and analysis suitability [46]. Our overall Kaiser–Meyer–Olkin value (measuring sampling adequacy of the correlation matrix) was 0.70, with all statements >0.6. We had responses to each statement from 120 handlers (110 mahouts, 10 head mahouts). In accordance with parallel analysis using the *paran* function (*paran* package; Dinno, 2012, [47]), the PCA reduced statements to 5 components with eigenvalues >1, accounting for 65% of the total variation. We set the threshold for statements loading onto components as 0.5, and all statements loaded onto a component.

If mahout attitudes towards their profession are changing as we hypothesise, it is vital to understand which traits underlie these attitudes and opinions. We therefore carried out further analysis into whether mahout and elephant characteristics correlated with the components. We used *glmer* models (*lme4* package [48]), with a gamma response distribution and an inverse link function, all numeric variables were scaled, and component scores centred to be positive (+2). Component scores were the response variable, fixed effects (depending on the model, see **S2 Table**) were job duration (continuous), number of elephants (integer), elephant age (continuous), elephant sex (binary: male/female), elephant behaviour as assessed by own mahout (binary: difficulties/ none), apprenticeship (binary: 1/0), and interviewer was a random effect (6 level factor).

## Results

### Expert questionnaires

**Fig 1:**
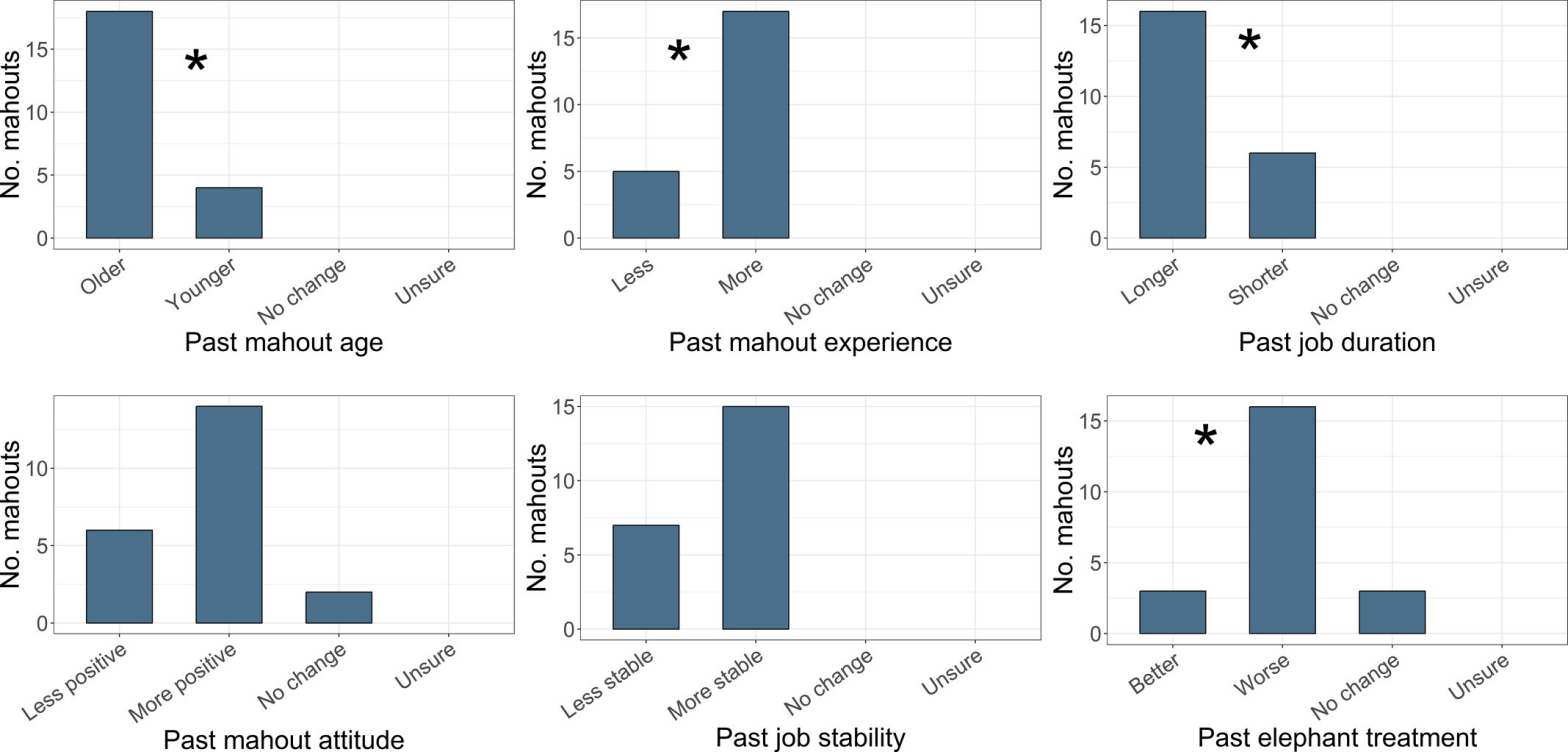
Expert’s observations of changes to elephant handling since they started working. The * symbol denotes statistical significance.

Experts were on average 38 years old (range 26–57), with 18 years of experience (10–39) and had perceived significant change since they began working (**Fig 1**). A significant majority (83%) thought mahouts used to be older (χ^2^ = 9.78_1_, *p*<0.01, n = 23), more experienced (78%) (χ^2^ = 7.35_1_, *p*<0.01, n = 23), and spent longer in the job (73%) (χ^2^ = 4.55_1_, *p*<0.05, n = 22). Although more experts (64%) thought past mahout job attitudes were more positive and the job more stable (68%), these were not significant majorities (χ^2^ = 3.2_1_, *p* = 0.074, n = 20; χ^2^ = 2.91_1_, *p* = 0.088, n = 22 respectively). A significant majority (73%) thought past elephant treatment was worse (χ^2^ = 8.89_1_, *p*<0.01, n = 19). When asked to elaborate on changes, experts reported “more techniques/ training/ care” currently. Perceived timing of changes ranged from 2000–2015, with a mode of 2013, and median 2011.

### Mahout questionnaires

On average, mahouts were 22 years old (range 14–59) and head mahouts 38 (27–59). Their 154 elephants were on average 15 years old (mean 24, range 4–72) and 56% were female. Mahouts had spent three years in their job on average (2 months-29 years), and head mahouts 19 years (6–43 years). Mahouts are expected to spend two years as an apprentice, to learn vital skills, yet only 35% and 17% of head mahouts and mahouts respectively had been an apprentice, for an average of two months for mahouts (2 weeks- 2 years) and 12 months for head mahouts (3 months-2 years). Mahouts had on average been working with their current elephant for 12 months (10 days-16 years), and had cared for two elephants (1–15) altogether in their career.

Investigating family connections to handling, 45% of mahouts and 65% of head mahouts had another handler in their family (son/ father/ brother/ uncle/ nephew/ grandfather/ in-law), with 16% of mahouts’ and 20% of head mahouts’ fathers having handled elephants.

#### Elephant behaviour

We found 65% of mahouts described their elephant as easy to handle, 27% as reasonable, and 8% as difficult (5% of females/ 11% of males). When asked for specific difficulties, the most common were ‘head shaking/ running/ difficult to collect from forest’ and there was little difference between the sexes (females: 20%; males: 22%). In terms of signals assumed in the literature [42,43] to indicate difficult behaviour (threatening/ agonistic), 30% of mahouts reported their elephant to ‘hit their trunk on the ground/ swing it at other elephants/mahouts’, 27% to ‘shake their head when approached’, and 13% displayed both. Many mahouts (42%) did not interpret these signals as expected (i.e. as indicative of difficult behaviour), instead reporting them to be signs of a “normal” or “friendly” elephant.

We explored factors influencing an elephant’s response to commands. We found 44% of elephants were reported to respond only to their own mahout, 35% to some others, and 21% to everyone. An elephant’s behaviour, a mahout’s command authority, and relationship length between mahout and elephant were all considered ‘*very important*’ by >95% mahouts. Elephant age and status (e.g. dominance) were rated ‘*very important*’ by 78% and 73% mahouts respectively. The use of tools (e.g. ear-hook) was considered ‘*very important*’ by 71% of mahouts and ‘*not important*’ by 22%. Due to acquiescence bias (a tendency to answer questions positively), these factors should be interpreted in relation to each other.

#### Job commitment and preferences

All head mahouts and 89% of mahouts claimed their job was a long-term commitment. However, of the 18 head mahouts and 65 mahouts with children, only 26% and 29% respectively thought their children would become a mahout too, suggesting commitment is not projected to their children’s generation. These responses were age-structured: 37% of older mahouts (aged 29–58, n = 34) thought their son would become mahout, compared to only 13% of younger mahouts (aged 19–28, n = 31). We next investigated mahout preference over their elephant’s sex, as bulls are traditionally preferred. Although 71% had no preference, 67% of those who did, preferred bulls (Chi-squared test: χ^2^ = 5.23_1_, *p*<0.05, n = 43). As bulls are generally more difficult to handle (less predictable and more prone to accidents [31,33]), we investigated whether this preference could be due to associated risk, asking whether mahouts would like to ride a difficult bull. Only 24% of mahouts answered ‘*yes*’, compared to 75% of head mahouts, suggesting preference was linked more to competency and experience, which is supported by those mahouts answering ‘*yes*’ having 3 years more experience on average.

#### Taming procedure

Elephant taming has received attention from welfare advocates in recent years for its perceived cruelty. We investigated taming from the point of view of the mahouts, as those people most involved in and most affected by its outcome. Of the 120 handlers (70%) who had witnessed it, 98% were positive towards traditional taming (the most common answers stating it helped calves be ‘obedient/ easier to handle/ clever’). However, when asked about their emotions during taming, 60% pitied the elephants but thought the process was necessary, 37% felt normal, 1% upset, and 3% were unsure. When questioned further, 36% would change the procedure, often to ‘be kinder/ use a softer method/ take more time to tame from a younger age’. When asked about using methods based on reward rather than punishment, 54% thought it possible on its own, 12% thought it sometimes possible on its own, and 34% did not. This latter minority mostly reasoned ‘traditional taming is best/ a combination of traditional and reward’ rather than specifically criticising reward-based taming. Handlers with more experience were significantly more likely to think reward-based taming would work, but their age did not have a significant effect (χ^2^ = 5.353, p<0.05, n = 122; χ^2^ = 0.00, p = 0.98, n = 122). Those in favour of reward-based taming assert that it may ‘take more time, but worth it if kinder/ mix of reward and traditional best’.

#### Mahout-elephant interactions

Final questions assessed handler agreement to 17 statements on the subject of elephants, reduced into 5 components using PCA. The first component (**Table 1)** loaded with statements about job attitudes (labelled as “job appreciation”), the second with those surrounding the importance of experience for being mahout (“experience is necessary”), and the third with those about elephant interest in humans (“human-elephant interaction”). The fourth component loaded with statements expressing experience and knowledge (“own knowledge”), and the fifth mostly with those relating to a mahout’s relationship with their elephant (“elephant relationship”), as well as their job.

**Table 1:** Principal component scores of 17 statements relating to elephant handling. Statements loading onto each component are shown in bold, with a loading limit of 0.50.

Statement	‘Job appreciation’	‘Experience is necessary’	‘Human-elephant interaction’	‘Own knowledge’	‘Elephant relationship’
You like working with elephants	**0.86**	0.08	0.05	0.01	0.28
You enjoy spending time with elephants	**0.81**	0.12	0.13	0.08	0.27
You will always be a mahout	**0.58**	-0.04	0.11	0.24	-0.17
Elephants are clever	**0.56**	0.50	-0.03	0.08	-0.31
You have a lot to learn about being mahout	0.05	**0.83**	0.07	0.11	0.04
Lots of experience is needed to be mahout	-0.03	**0.81**	0.08	0.17	0.04
You like working with other mahouts	0.18	**0.68**	0.16	-0.08	0.17
Elephants are friendly	0.13	0.08	**0.84**	0.06	0.06
Elephants are obedient	-0.02	0.05	**0.81**	0.25	0.13
Elephants like to socialise with people	0.23	0.08	**0.66**	-0.15	0.06
Elephants respond to you talking to them	-0.09	0.41	**0.53**	0.26	0.04
You have a lot of experience with elephants	-0.02	0.07	0.04	**0.83**	0.04
You know a lot about elephant health	0.34	0.30	0.03	**0.71**	0.10
You understand your elephant’s behaviour	0.10	0.00	0.15	**0.69**	0.15
You are patient with your elephant	0.07	0.10	0.11	0.08	**0.82**
You & your elephant have a special bond	0.01	0.08	0.03	0.29	**0.64**
Mahout is a good job	0.49	-0.02	0.18	-0.11	**0.60**
Eigenvalue	4.35	1.99	1.76	1.59	1.28
Variance (%)	15	14	13	12	11

We next investigated how factors such as a mahout’s experience in elephant handling or their elephant’s age, sex and behaviour influenced mahout answers. We found no traits significantly correlated to either “job appreciation” or “elephant relationship” (model outputs in **S2 Table**). Experienced mahouts agreed more that “experience is necessary” for the job (t = 2.44, n = 93, *p*<0.05), as did mahouts of bulls (t = 2.47, n = 93, *p*<0.05) and younger elephants (t = -2.05, n = 93, *p*<0.05). Mahouts of difficult elephants scored lower for “human-elephant interaction” (t = -2.59, n = 89, *p*<0.05). Finally, the longer a mahout had worked with elephants (t = 2.39, n = 97, *p*<0.05), and the more elephants he had cared for (t = 2.04, n = 97, *p*<0.05), the greater his “own knowledge” component.

## Discussion

The mahout profession is in decline across Asia, due to the reduced economic and cultural importance of elephants, as reported by Lair over two decades ago [24]. These changes are particularly pronounced in more industrialised countries such as Sri Lanka, India and Thailand, whereas Myanmar is often quoted to be one of the last strong-holds of traditional mahouts [24,49]. In line with such change, Indian mahouts have been found to spend less time with and frequently switch elephants following reduced employment opportunities [34]. According to the experts in this study, who cumulatively had 429 years of experience and therefore harbour invaluable knowledge, current mahouts in Myanmar are less experienced and spend less time in the job than in the past. These changes could threaten the mahout profession, that has traditionally relied on long-term relationships and commitment [20]. Interestingly, however, experts perceived current elephant treatment to be better than in the past, describing “more techniques, training and care” available currently. As less than a quarter of experts were mahouts, these “techniques” may concern management or veterinary care rather than handling. One answer stated “although techniques are better now, mahouts do not follow them”, suggesting improvements may not be reaching mahouts, perhaps as they change too often for techniques to be learnt and passed on. Experts reported changes to have occurred in 2011/13 (median/mode), which correspond with major political and social change in Myanmar [50]. These changes made technologies such as mobile phones and mopeds easier and cheaper to access, improving communication and access to remote areas [51,52]. Such changes are likely impacting the mahout profession, with competing jobs, such as factory work, more accessible to rural communities [27].

Experts also perceived current mahouts to be younger, which is at odds to the ageing mahout population reported in the Lao logging industry, but which mirrors the young population of mahouts in the Lao tourism industry [30]. The difference is likely due to the lower employment opportunities for timber elephants and their mahouts in Laos compared to Myanmar, leading boys from Lao mahout families to avoid the profession entirely [30]. Currently, the problem in Myanmar lies in retaining long-term mahouts, but as logging reduces in Myanmar- exemplified by the ban of round-log export in 2014 [53]- similar recruitment issues need to be anticipated in the future and alternative options for timber elephants considered. However, younger mahouts may also offer some advantage to their elephants: older mahouts have been reported to tire of their elephants over time, and spend less time with them [34]. Further investigation is therefore required into how mahout age and experience impact their elephants’ well-being to fully understand the implications of change.

Our survey of current mahouts employed in the Myanma timber industry was in line with the experts’ observations; they were young and had little experience. This is important, because temporal pessimism (tendency to remember the past positively [54]) or skewed comparison (e.g. remembering past mahouts as older as interviewees were younger then) may have introduced bias to expert perceptions, which makes it necessary to supplement these observations with data-driven assessments of current mahouts. The results from this survey contrast the traditional system of apprenticeship-based learning and life-long mahout-elephant relationships, maintained at least in parts of India [35]. These results highlight potential differences between countries, but may contrast the common assumption that Myanmar has retained the most traditional mahouts [49].

The majority of mahouts in our study skipped an apprenticeship period, which may be driven by a shortage of mahouts or reticence to invest in an undesirable job, but which jeopardises mahout and elephant safety. A study by Vanitha et al. [33] highlighted the risks of deviating from the traditional system, with loss of traditional mahouts coupling with more fatal accidents in temples, and Radhakrishnan et al. [32] exposed the severity of risks for mahouts. The mahouts in our study change elephants almost yearly, which is concerning considering Srinivasaiah et al. [34] suggest three years is needed to understand an elephant’s behaviour, and eight years to develop trust. This issue is complicated by almost half of our elephants reported to respond only to their own mahout, which has also been described in past studies [26], suggesting there may be a period of adjustment when mahouts change. Future studies should investigate how frequent mahout change and mahout inexperience impact their elephants and whether they pose a threat to mahout safety.

Though male elephants tend to be less predictable in their behaviour, especially during musth, they have traditionally been preferred by logging mahouts for their strength and status [31]. Lair [24] hypothesised one major indicator of mahout-ship decline would be a diminished ability to handle bulls, which may be seen in the general preference for docile females found in tourism camps across Asia, leading to severely female biased sex ratios [30,55]. Our study still found mahouts to have a preference for bulls, despite more bulls being described as difficult, which may suggest a certain knowledge of handling bull behaviour, especially present in head mahouts and experienced mahouts. However, many mahouts interpreted behaviours assumed in the literature to be “agonistic” or “threatening” as “normal” or “friendly” which could indicate lack of behavioural understanding, although it is also possible that these behaviours are simply part of the normal behavioural repertoire in the study population.

The second area of elephant handling proposed by Lair [24] to indicate mahout-ship decline, was a reduced ability to tame elephants. Our finding that most handlers had been involved in the taming process contrasts other countries where many younger mahouts inherit already tamed elephants and show little interest in learning the necessary skills. Traditional elephant taming has been a contentious topic in the media and among welfare advocates in recent years, with pressure to adopt methods based on positive rather than negative reinforcement [56]. It is also a critical issue in this population, with high mortality during the taming ages of 4–5 years (see [54]). However, there are major risks to consider when proposing changes to taming in semi-captive working populations [32], so we explored mahout willingness to accept changes to traditional taming, as those people most integral to the process and most influenced by changes [57]. Their responses showed promise for future progression towards positive reinforcement, as most expressed pity for the elephants, and support for reward-based taming at least some of the time. Interestingly, experienced mahouts were more likely to think reward-based techniques were possible to train elephants. Handlers often expressed concern for the greater time and money required for novel, reward-based taming- essential issues to be considered in future discussions surrounding taming management.

Another trend seen across Asia is a lessening traditional family connection to elephant handling, as the job becomes less appealing to younger generations [30]. The extent of this change differs between countries and regions [34,56]. We found most of the head mahouts and almost half of the mahouts in our study had a family connection to handling, but few wished their sons to become mahout too, reflecting sentiments found in parts of Thailand and India [24,32]. This was particularly accentuated in mahouts under 30, suggesting further decline in the future, which is troubling if the aforementioned risks of deviating from the traditional system are true (see [32,33]) and reinforces the need to understand how the length and stability of the mahout-elephant relationship affect elephant welfare.

In such a time of transition and change within the mahout profession, it is necessary to explore any factors underpinning mahout attitudes towards their profession. The factors we found to influence mahout attitudes again reflect the two indicators outlined by Lair [24], with mahouts of bulls and younger elephants more likely to agree that “experience is necessary” and those with more difficult elephants scoring lower for “human-elephant interaction". These issues could be alleviated by increased understanding of difficult behaviour, particularly during juvenile years and musth (in accordance with Srinivasaiah *et al*., [34]).

## Conclusions

Overall, our study suggests major differences between the assumed traditional mahout system and that of the largest semi-captive global population of Asian elephants. Shifts are mirroring patterns of change across Asia seen hitherto mostly within the tourism industry and more industrialised countries [30,58]. We found current mahouts in Myanmar have little experience, are quick to change elephants, and often lack familial connection to the profession. We propose the mahout system may have to adapt to offer more formal training and incentives for mahout recruitment and retention, consistent with past recommendations for elephant welfare [29,56,59], and an argument generally applicable to one third of this endangered species in similar conditions [59].

## Supporting information

S1 Table Number of responses given for each question in both the expert (E) and mahout (M) questionnaires and answer options where applicable. Question style is denoted as O (Open ended), M (Multiple choice) or L (Likert scale).
(DOCX)

S2 Table Effect of mahout and elephant characteristics on PCA components (i) ‘Job appreciation ‘; (ii) ‘Experience is necessary’; (iii) ‘Human-elephant interaction’ (iv) ‘Own knowledge’ and (v) ‘Relationship with elephant’. * shows statistical significance and—shows terms missing from final model. Sample sizes are as follows: (i) n = 89 (ii) n = 93 (iii) n = 89, (iv) n = 97, (v) n = 89.
(DOCX)

S1 Dataset File containing data from mahout questionnaires. (CSV)

S2 Dataset File containing data from expert questionnaires. (CSV)
