# Supplementary material for: Investigating changes within the handling system of the largest semi-captive population of Asian elephants
Source: PLoS One. 2019 Jan 31;14(1):e0209701. doi: 10.1371/journal.pone.0209701 (PMC6354975; doi:10.1371/journal.pone.0209701)
Supplement: S1 Table — Question style is denoted as O (Open ended), M (Multiple choice) or L (Likert scale). (DOCX) [file pone.0209701.s001.docx]

**S1 Table. Number of responses given for each question in both the expert (E) and mahout (M) questionnaires and answer options where applicable.**

| Question | Number  responses | | Question  style | | Answer options |
| --- | --- | --- | --- | --- | --- |
| E.1 How many years have you worked in MTE? | | 23 | | O | NA |
| E.2 What is your job in MTE? (eg.vet/ mahout/ sin oke) | | 23 | | O | NA |
| E.3 Name | | 23 | | O | NA |
| E.4 Age | | 23 | | O | NA |
| *Do you notice any differences (compared to current mahouts) in terms of …* | | | | | |
| E.5...Average mahout age when you started working? | | 23 | | M | Younger/Older/No change/ Unsure |
| E.6...General mahout experience level when you started working? | | 23 | | M | More experienced/Less experienced/No change/Unsure |
| E.7...Length of time mahouts spent in the job when you started working? | | 22 | | M | Longer/shorter/No change/Unsure |
| E.8...General (mahout) attitude towards the job when you started working? | | 22 | | M | More positive/More negative/No change/Unsure |
| E.9...Stability of (mahout) job when you started working? | | 22 | | M | More stable/Less stable/No change/ Unsure |
| E.10...General treatment/ well-being of elephants when you started working? | | 22 | | M | Better/Worse/No change/Unsure |
| E.11 Please explain any other differences between working in the past and now | | 22 | | O | NA |
| E.12 When do you think the changes occurred? | | 17 | | O | NA |
| M.1 Handler name | | 210 | | O | NA |
| M.2 Handler age | | 210 | | O | NA |
| M.3 When did you become a mahout? | | 208 | | O | NA |
| M.4(a) Did you work as pae cheik (apprentice)? | | 176 | | M | Yes/No |
| M.4(b) If yes, how long did you train as pae cheik? | | 32 | | O | NA |
| M.5 How many elephants have you worked with in total? | | 208 | | O | NA |
| M.6 Do you prefer to ride male or female elephants? | | 170 | | M | Male/Female/No preference |
| M.7 Would you want to ride a dominant bull, known to be difficult? | | 167 | | M | Yes/No/Don’t know |
| M.8 How do you see this job? | | 170 | | M | Long term/Short term/Depends on other options |
| M.9 How many members of your family (and who) are handlers (sin-gaung/ mahout/ apprentice)? | | 206 | | O | NA |
| M.10 Are you married? | | 173 | | M | Yes/No |
| M.11 How many children do you have? | | 173 | | O | NA |
| M.12 If you have children, do you think they will be a mahout too? | | 82 | | M | Yes/No |
| M.13(a) Have you been involved in the taming process? | | 172 | | M | Yes/No |
| M.13(b) If yes, how many times? | | 120 | | O | NA |
| M.14(a) Do you think traditional taming is good for the elephants? | | 143 | | M | Yes/No |
| M.14(b) Why? | | 114 | | O | NA |
| M.15(a) Would you change anything about the traditional taming process? | | 138 | | M | Yes/No |
| M.15(b) Why? | | 50 | | O | NA |
| M.16 How do you feel during traditional taming when the calves are in the breast band? | | 142 | | M | Upset/Pity, but it’s necessary/Normal/Unsure |
| M.17(a) Do you think it's possible to tame using only reward, no punishment? | | 123 | | M | Yes/No/Sometimes |
| M.17(b) Why? | | 70 | | O | NA |
| *How much do you agree with the following statements …* | | | |  |  |
| M.18(a)...Elephants are obedient | | 171 | | L | 1-5: Definitely(1)/Quite(2)/Unsure(3)/Not really(4)/Definitely not(5) |
| M.18(b)...Elephants are friendly | | 170 | | L | 1-5 |
| M.18(c)...Elephants are responsive | | 136 | | L | 1-5 |
| M.18(d)...Elephants are interesting | | 170 | | L | 1-5 |
| M.18(e)...You like working with elephants | | 169 | | L | 1-5 |
| M.18(f)...You know a lot about elephant health | | 171 | | L | 1-5 |
| M.18(g)...You like working with other mahouts | | 169 | | L | 1-5 |
| M.18(h)...Elephants are dangerous | | 172 | | L | 1-5 |
| M.18(i)...Elephants like to socialise with people | | 172 | | L | 1-5 |
| M.18(j)...Being a mahout is a good job | | 171 | | L | 1-5 |
| M.18(k)...Elephants are clever | | 172 | | L | 1-5 |
| M.18(l)...You are patient with your elephant | | 172 | | L | 1-5 |
| M.18(m)...Lots of experience is needed to be a mahout | | 170 | | L | 1-5 |
| M.18(n)...Elephants are beautiful animals | | 166 | | L | 1-5 |
| M.18(o)...Elephants can feel a lot of pain | | 170 | | L | 1-5 |
| M.18(p)...You enjoy spending time with elephants | | 171 | | L | 1-5 |
| M.18(q)...You have a lot of experience with elephants | | 169 | | L | 1-5 |
| M.18(r )...You have a lot to learn about being a mahout | | 171 | | L | 1-5 |
| M.18(s)...You understand your elephant’s behaviour | | 170 | | L | 1-5 |
| M.18(t)...You’d like to learn more about elephants | | 169 | | L | 1-5 |
| M.18(u)...You have a special bond with your elephant | | 171 | | L | 1-5 |
| M.18(v)...You will always be a mahout | | 161 | | L | 1-5 |
| M.19 What is your present elephant’s ID number | | 191 | | O | NA |
| M.20 What is your present elephant’s date of birth | | 191 | | O | NA |
| M.21 What is your present elephant’s sex | | 190 | | M | Male/Female |
| M.22 How long have you worked with this elephant? | | 188 | | O | NA |
| M.23 How easy is your elephant to control | | 187 | | M | Easy/Reasonable/Difficult |
| M.24(a) Do you experience difficulties with this elephant? | | 110 | | M | Yes/No |
| M.24(b) If yes, what are they? | | 34 | | O | NA |
| M.25 Does your elephant respond to commands given by other mahouts or only to you? | | 151 | | M | Only own mahout/Some others/Everyone |
| M.26 Does your elephant hit the ground with its trunk/ swing it at other elephants/ mahouts | | 167 | | M | Yes/No |
| M.27 Does your elephant ever shake its head when approached? | | 166 | | M | Yes/No |
| M.28 What does your elephant's behaviour (in M.26/27) indicate? | | 162 | | M | Difficult/Normal/Friendly/Unsure |
| *How important are the following to the way your elephant responds to commands? ...* | | | | | |
| M.29(a)...Relationship length between the mahout giving command and elephant | | 118 | | M | 1-4: Very important(1)/Reasonably important(2)/Not important(3)/Unsure(4) |
| M.29(b)...Behaviour of the elephant | | 120 | | M | 1-4 |
| M.29(c)...Social status of elephant | | 120 | | M | 1-4 |
| M.29(d)...Use of tools (e.g. ankush) | | 121 | | M | 1-4 |
| M.29(e)...Age of elephant | | 122 | | M | 1-4 |
| M.29(f)...Authority of command | | 122 | | M | 1-4 |

Question style is denoted as O (Open ended), M (Multiple choice) or L (Likert scale)
