# Supplementary material for: Investigating changes within the handling system of the largest semi-captive population of Asian elephants
Source: PLoS One. 2019 Jan 31;14(1):e0209701. doi: 10.1371/journal.pone.0209701 (PMC6354975; doi:10.1371/journal.pone.0209701)
Supplement: S2 Table — * shows statistical significance and—shows terms missing from final model. Sample sizes are as follows: (i) n = 89 (ii) n = 93 (iii) n = 89, (iv) n = 97, (v) n = 89. (DOCX) [file pone.0209701.s002.docx]

**Table S2. Effect of mahout and elephant characteristics on PCA components (i) ‘Job appreciation ’; (ii) ‘Experience is necessary’; (iii) ‘Elephant agreeableness’ (iv) ‘Own knowledge’ and (v) ‘Relationship with elephant’.**

|  | (i) |  | (ii) |  | (iii) |  | (iv) |  | (v) |  |
| --- | --- | --- | --- | --- | --- | --- | --- | --- | --- | --- |
| Fixed effects | Estimate  ± SE | t-value | Estimate  ± SE | t-value | Estimate  ± SE | t-value | Estimate  ± SE | t-value | Estimate  ± SE | t-value |
| (Intercept) | 1.02 ± 0.14 | 7.56* | 1.07 ± 0.15 | 7.38* | 1.46 ± 0.20 | 7.25* | 0.78 ± 0.11 | 7.30* | 1.04 ± 0.19 | 5.59* |
| Years as mahout | 0.12 ± 0.09 | 1.35 | 0.19 ± 0.08 | 2.44* | -0.08 ± 0.07 | -1.10 | 0.31 ± 0.13 | 2.39* | 0.05 ± 0.11 | 0.46 |
| Behaviour (diff) | 0.02 ± 0.12 | 0.21 | - | - | -0.23 ± 0.09 | -2.59* | - | - | -0.02 ± 0.15 | -0.10 |
| Ele sex (Male) | -0.00 ± 0.08 | -0.01 | 0.18 ± 0.07 | 2.47* | - | - | - | - | 0.04 ± 0.11 | 0.36 |
| Ele age | 0.02 ± 0.08 | 0.21 | -0.13 ± 0.06 | -2.05* | -0.09 ± 0.07 | -1.31 | - | - | 0.11 ± 0.12 | 0.92 |
| No. elephants | - | - | - | - | - | - | 0.38 ± 0.19 | 2.04* | - | - |
| Apprentice (Yes) | - | | -0.16 ± 0.11 | -1.41 | - | - | 0.11 ± 0.16 | 0.72 | - | - |
| Random effects | Variance  ± SD | | Variance  ± SD | | Variance  ± SD |  | Variance  ± SD |  | Variance  ± SD |  |
| Interviewer | 0.04 ± 0.19 | | 0.03 ± 0.17 | | 0.06 ± 0.25 |  | 0.01 ± 0.08 |  | 0.04 ± 0.19 |  |
| Residual | 0.20 ± 0.44 | | 0.11 ± 0.33 | | 0.14 ± 0.37 |  | 0.18 ± 0.43 |  | 0.18 ± 0.42 |  |

* shows statistical significance and - shows terms missing from final model. Sample sizes are as follows: (i) n=89 (ii) n=93 (iii) n=89, (iv) n=97, (v) n=89.
